# A Rare Case of a Migrating Inguinal Hernia Mesh Presenting as Acute Appendicitis

**DOI:** 10.1155/2021/2007935

**Published:** 2021-09-14

**Authors:** Jorge Nogueiro, Hugo Santos-Sousa, Marinho de Almeida, Luis Malheiro, Elisabete Barbosa

**Affiliations:** Department of General Surgery, São João University Hospital Center, Porto, Portugal

## Abstract

Acute appendicitis is a very common event. Migration of hernia mesh is rare, especially intraluminal migrations. We aim to report a case of a migrated inguinal mesh presenting as an acute appendicitis. A 58-year-old male previously submitted to ONSTEP right inguinal hernia repair with a PolySoft™ hernia patch eight years before, was admitted in the emergency department with acute appendicitis, and submitted to laparoscopic appendectomy. Intraoperatively, the “recoil ring” from the inguinal hernia patch was extended from the anterior abdominal wall to the appendix, perforating it and progressing intraluminally. Appendectomy was performed, as well as removal of the mesh by an anterior approach. Hernia mesh migration to an intraluminally position is extremely rare with only a few cases described in literature. Pathogenesis of migration is still poorly understood. Clinicians should consider hernia mesh migration in their differential diagnosis for causes of acute appendicitis, in the right clinical setting, when a previous hernia defect correction is present. To the best of our knowledge, this is the first reported case of inguinal hernia mesh migration to the appendix, presenting as acute appendicitis.

## 1. Introduction

Acute appendicitis (AA) is one of the most common conditions presenting at the emergency department in daily practice. Prompt diagnosis is mandatory and, in the majority of the cases a surgical procedure is required. Although several causes of AA are possible, it is not always clear why certain patients develop this condition [1] and sometimes during the surgery one can find rare causes of AA. A detailed medical history is essential to understand the cause behind AA in each patient.

We report a case of a migrated inguinal mesh presenting as AA. This report has received approval by the local Ethics Committee.

## 2. Case Report

A 58-year-old male was admitted in the emergency department of our Institution complaining of diffuse, intense and worsening abdominal pain migrating to the right iliac fossa, with eight hours of duration. The patient denied nausea, vomiting, or altered bowel movements. The patient had a relevant medical history of hypertension (medicated with Perindopril 4 mg 1id). He had been previously submitted to ONSTEP right inguinal hernia repair with a PolySoft™ hernia patch [2] eight years before, also at our Institution, and apparently there wasn't any sign of recurrence. He had no other relevant medical or surgical history.

On physical examination the patient presented an auricular temperature of 37.8°C and was hemodynamically normal. He experienced pain in the right inferior quadrant of the abdomen on palpation, with a Blumberg and Rovsing positive signs. No hernias were noted. Blood tests showed elevated white blood count with increased neutrophils, along with elevated C reactive protein, without other relevant alterations. Urinalysis was unremarkable. A diagnosis of acute appendicitis was suspected, and an abdominal ultrasound was done, which revealed a luminal dilation of the appendix with 10 mm diameter, which was non-compressible, along with echogenic, prominent peri-appendiceal fat, and a thin layer of peri-appendiceal fluid, suggestive of acute appendicitis. Intravenous antibiotics and a subsequent laparoscopic appendectomy were proposed.

**Figure 1:**
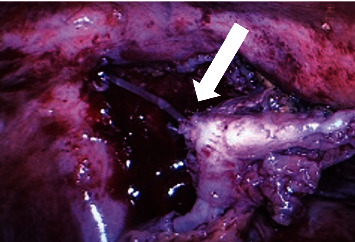
Laparoscopic view of suppurated appendix with the “recoil ring” migrated intra-luminally (arrow).

Intraoperatively, the appendix was highly adherent to the anterior abdominal wall, reddish, dilated and with evidence of suppuration with small amount of free fluid. During the dissection, a foreign body was detected, clearly originating from the anterior abdominal wall. This foreign body, unequivocally perforated the appendix and progressed intraluminally. The material was noticed to be the recoil “ring” from the inguinal hernia patch previously applied to the patient for treating his inguinal hernia (Figure 1). Appendectomy was then performed. Purulent fluid was released from the anterior abdominal wall and infection of the mesh was noticed; in this line, removal of the non-migrating remaining mesh by an anterior approach was subsequently performed. An intraperitoneal drain was left directed to the right iliac fossa.

Histologically, acute appendicitis with extensive serositis was confirmed. The drain was removed at day two post-operatively, the post-operative course was unremarkable and the patient was discharged three days after the procedure.

## 3. Discussion

Surgical repair of abdominal wall hernia is amongst the most common surgical procedures performed worldwide in Surgical Departments, with around 20,000,000 groin hernia repairs performed annually [3]. Despite the variety of techniques for correcting these abdominal wall defects, the gold standard approach is definitely the use of a mesh in repair, which significantly reduces the incidence of recurrence. However, complications such as infections and chronic pain do exist. Mesh migration, although less frequent, is also a well-known complication [4]. It has been documented in a wide array of situations, including after correction of inguinal, umbilical, ventral and incisional hernias, and with a variable post-surgical timing, from months to years after the procedure [4]. In a comprehensive review, Cunningham et al. reported 89 cases of hernia mesh migration. This was predominant in male patients (76.2% of the cases) and the average patient age was 59.8 years. In this review 56 cases of inguinal hernia mesh migration were reported between 1996 and 2017 [4].

As mesh migration can occur with a wide variety of organs involved, including colon, small bowel or bladder, clinical presentation of these patients varies according to the site affected [5]. To the best of our knowledge, this is the first reported case of inguinal hernia mesh migration to the appendix, presenting as AA. The migration of the mesh to appendix lumen was probably responsible for the AA as it might have obstructed the appendiceal lumen.

The event of migrating hernia mesh is still not completely understood. Literature suggests that mesh fixation may interfere with this event but no sufficient and reliable data is available that allows a strong conclusion. On the other hand, mesh fixation may related to postoperative pain and nerve entrapment. For this reason it is still not clear what the best approach is to avoid this complication.

Removal of the mesh was challenging. The presence of suppuration in the abdominal wall determined the option to go for an anterior approach to the right inguinal area, leading to a complete removal of the prothesis. The patient is currently in follow-up for prompt identification of hernia recurrence, but after 2 years has not shown evidence of this.

## 4. Conclusion

With the increasing popularity of mesh repair of abdominal hernias, even though mesh migration is an uncommon complication, we could expect for an increasing number of patients presenting with such scenario. Given the non-specific symptoms and wide array of possible clinical presentations, the key for early identification of this potentially severe complication is simply the awareness of its existence.

Hence, clinicians should consider this in their differential diagnosis for causes of AA, in the right clinical setting, when a previous hernia defect correction is present.
